# Adequacy of iodine intake in three different Japanese adult dietary patterns: a nationwide study

**DOI:** 10.1186/s12937-015-0116-y

**Published:** 2015-12-30

**Authors:** Ryoko Katagiri, Keiko Asakura, Ken Uechi, Shizuko Masayasu, Satoshi Sasaki

**Affiliations:** Department of Social and Preventive Epidemiology, Graduate School of Medicine, the University of Tokyo, 7-3-1 Hongo, Bunkyo-ku, Tokyo, 113-0033 Japan; Department of Social and Preventive Epidemiology, School of Public Health, the University of Tokyo, 7-3-1 Hongo, Bunkyo-ku, Tokyo, 113-0033 Japan; Interfaculty Initiative in Information Studies, the University of Tokyo, 7-3-1 Hongo, Bunkyo-ku, Tokyo, 113-0033 Japan; Ikurien-naka, 3799-6 Sugayahorinouchi, Naka-city, Ibaraki Japan

**Keywords:** Iodine, Dietary patterns, Cluster analysis, Iodine adequacy, Japanese

## Abstract

**Background:**

Iodine intake is considered to be high in Japan due to regular seaweed consumption. Subgroups that do not have a traditional Japanese-style diet may consume insufficient amounts of iodine.

**Method:**

Three hundred and ninety apparently healthy adults (195 men and 195 women) aged 20 to 69 years from 20 areas throughout Japan completed four-day diet records and collected a 24-h urine sample. Dietary patterns were extracted from 31 food groups by cluster analysis. The iodine adequacy of each dietary pattern was examined using reference values from the Dietary Reference Intakes for Japanese.

**Results:**

Three dietary patterns, labelled “Cluster I (Rice and vegetables)” (*n* = 101), “Cluster II (Meat, non-Japanese noodles, and sugar-sweetened beverages)” (*n* = 34), and “Cluster III (Fish, Japanese noodles, and alcohol)” (n = 60), were identified in male subjects. Another set of three patterns, “Cluster I (Rice and vegetables)” (*n* = 22), “Cluster II (Fish and Japanese noodles)” (*n* = 33), and “Cluster III (Bread and non-Japanese noodles)” (*n* = 140), was found in female subjects. Although the habitual iodine intake of almost all participants was above the estimated average requirement (EAR), iodine intake was statistically significantly lowest in Cluster II in men and Cluster III in women. Moreover, the mean participant age was the youngest in these clusters.

**Conclusion:**

Although Japan is known as a high iodine-consuming country, some Japanese individuals who do not eat a traditional Japanese-style diet consume low amounts of iodine. Since younger people tend to have modern, Westernized dietary patterns, iodine deficiency might be given additional consideration hereafter in Japan.

**Electronic supplementary material:**

The online version of this article (doi:10.1186/s12937-015-0116-y) contains supplementary material, which is available to authorized users.

## Introduction

A dietary pattern approach, in which the effect of a combination of foods can be evaluated, has been developed [1]. Associations between dietary patterns and health outcomes have been demonstrated through this approach [2]. The nutritional adequacy of dietary patterns calculated from a comparison of nutrient intake with Dietary Reference Intakes or the World Health Organization’s recommended values have also been evaluated in some studies [3–6]. These studies have mainly been conducted in Western countries [3, 4], although Okubo et al. assessed the nutritional adequacy of four dietary patterns identified by cluster analysis in young Japanese women [5] and three dietary patterns in pregnant Japanese women [6]. These studies employed cluster analysis, which is a posteriori method used to derive eating patterns that is common in factor analysis. Cluster analysis can create patterns that represent relatively homogeneous dietary intake groups and can divide subjects into clusters, thus allowing the differences in nutritional status among clusters to be evaluated [1]. Macronutrients and several vitamins and minerals were assessed in these previous Japanese studies [5, 6]; however, iodine, which is not completely listed for all food items in the Standard Composition Table in Japan 2010 [7], was not examined.

Iodine deficiency is a worldwide public health problem [8], while iodine is a unique nutrient that should be evaluated in Japan as a wide range of iodine intake has been observed in the Japanese population [9]. The main food source of iodine in Japan is seaweed, especially kelp and soup stock made from kelp [10]. The iodine intake of the older generation who consume more seaweed is generally higher than that of the younger generation [10]. The median habitual iodine intake was shown to around 850 to 1000 μg/day and a review estimated that the iodine intake for Japanese individuals was 1000 to 3000 μg/day [9, 10]. According to the Dietary Reference Intakes 2015 for Japanese (DRI) [11], the tolerable upper intake level (UL) is set as 3000 μg/day in Japan, which is higher than that of other countries (e.g., the UL for American adults is 1100 μg/day). The habitual iodine intake of almost 10 % of participants in a previous Japanese study exceeded the Japanese UL [10]. The excess intake assessed by urine samples is associated with subclinical hypothyroidism [12]. Despite the possibility of excess iodine intake in the general Japanese population, the cut-off value that causes apparent clinical symptoms has not yet been fully determined in the Japanese population [11]. In contrast, although dietary iodine deficiency has not been focused on in Japan, some Japanese individuals, especially those in the younger generation such as students, consume less iodine than the recommended dietary allowance (RDA) in Japan (130 μg/day) [13]. Therefore, both excess and insufficient iodine intake warrant further investigation. Seaweed and soup stock made from seaweed, which are the main sources of iodine in Japan [10], are used in many Japanese dishes (e.g., miso soup, noodles, and processed foods). As such, it is considered helpful to identify the adequacy of iodine intake in typical dietary patterns. A single-food approach might not enable us to precisely determine the reason for inadequate iodine intake due to the complexity of iodine content in dishes consumed in Japan.

Thus, in the present study, we extracted dietary patterns from current Japanese diets and examined the adequacy of iodine intake for each pattern by comparing the intakes estimated by the diet records (DR) and the urinary excretion of iodine with the DRI.

## Methods

### Study population

In February and March 2013, apparently healthy Japanese individuals aged 20 to 69 years from 20 areas throughout Japan were recruited. The details of this survey were described elsewhere [14]. Briefly, 199 dietitians (called research dietitians) throughout Japan working at welfare facilities recruited their co-workers; in each area, 40 subjects were expected to participate in the survey. The 40 participants consisted of eight subjects (four men and four women) in each of five 10-year age classes (20–29, 30–39, 40–49, 50–59, and 60–69 years). Overall, 791 subjects (395 men and 396 women) participated in this survey and were asked to collect a 24-h urine sample. To ensure feasibility, approximately half of the study participants (*n* = 392, approximately 20 subjects from each study area (two men and two women in each of the age ranges)) were asked to complete a 4-nonconsecutive-day DR.

### Ethics, consent and permissions

Research dietitians explained the purpose and protocol of this survey to the participants individually and each participant completed an informed consent form to participate. The ethics committee of the University of Tokyo, Faculty of Medicine approved all procedures of this survey (No. 10005).

### Dietary assessment

A 4-day semi-weighed DR was utilized for the study. Participants were asked to record their diets for 4 non-consecutive days (three working days and one day off, excluding days before and after night work). Consumed foods (ingredients and prepared dishes) and drinks were weighed with a provided digital kitchen scale (KD-812WH, Tanita, Tokyo, Japan) or measured by a measuring cup or a measuring spoon. When weighing was difficult, the names of dishes and restaurants and whether there were any leftovers were recorded. From this information, food weight was estimated instead of measuring weight with the provided scale. Recording sheets were submitted to research dietitians and were immediately checked by them and two research dietitians at the central office of this study. Iodine intake was calculated using the previously developed food composition table which was compensated the Standard Table of Food Composition in Japan 2010 [7, 10]. Salt iodization is not implemented in Japan.

### 24-hour urine collection and other variables

24-hour urine was collected by all participants who completed the DR (*n* = 392). Details of the urine collection method were described elsewhere [14]. Briefly, the iodine in the 24-h urine samples was measured once. Collected urine was measured by LSI Medience Corporation (Tokyo, Japan). Urinary iodine was measured using an iodine measurement kit (Hitachi Chemical Co., Ltd., Tokyo, Japan). In this kit, urinary iodine concentration was assessed on a microplate using the Sandell-Kolthoff reaction. Then, colorimetric measurement at 405 nm was conducted with microplate reader “IMMUNO-MINI NJ-2300” (Biotec Company Limited, Tokyo, Japan) [15]. Creatinine concentration was measured by JCA-BM6050 (JEOL Limited, Tokyo, Japan), which employs the enzyme method.

A questionnaire was used to inquire about the background of participants, including smoking habits, marital status, past medical history, and medications. Body height was measured to the nearest 0.1 cm and body weight was measured to the nearest 0.1 kg with light clothing. Research dietitians or medical workers in the welfare facilities measured these anthropometric values. Body mass index (BMI) was calculated as body weight (kilograms) divided by the square of body height (meters).

### Food grouping

Food items in the DR were coded according to the food codes of the Standard Table of Food Composition in Japan 2010 [7]. These items were classified into 31 food groups based on the classification of the standard composition table [7], similarity of nutrition, and classification used in previous studies [16]. These groups are shown in Additional file 1: Table S1. When food items were written in dried-weight in DR, they were changed into the consumed (wet) amount, consistent with the previous paper [16]. Flour not in bread or confectionaries, corns and wheat gluten were impossible to assign to the pre-decided food groups and were excluded from the analysis. Soup stock and miso (fermented soybean paste) were divided and classified into Japanese noodles, other noodles (e.g., Chinese noodles), miso soup, and seasonings according to their usage.

Although several formats for food group variables, such as servings, grams, or the percentage total energy contribution from food exist, the best method has not yet been determined [17, 18]. In the present study, energy-adjusted amounts of food using the density method (g/1000 kcal) were selected to conduct cluster analysis that allows comparison with previous studies [5, 6], and the interpretation of clusters. Then, the amount of energy-adjusted food groups was standardized to a mean of zero and a standard deviation of one, consistent with previous studies.

### Data analysis

Of the 392 participants who completed DRs, participants who had a medical history of thyroid disease (*n* = 1, one woman) and one man who drank over 1 liter of fruit and vegetable juice per day were excluded because the former might have altered dietary habits relating to iodine and the latter was an extreme outlier from other participants. Other participants were not excluded from the evaluation of DR because energy intake of them was not too low (<500 kcal/d) or too high (>4000 kcal/d). Cluster analysis and the comparison of intake with DRI were performed for these 390 participants (195 men and 195 women). To confirm the completeness of the 24-h urine collection, the ratio of observed to expected creatinine excretion was used according to previous studies [14]. When the ratio was < 60 % or > 140 %, participants were excluded from the analysis of excretion (*n* = 34).

Cluster analysis was performed using PROC FASTCLUS in SAS 9.4 (SAS Institute Inc., Cary, NC, USA). The K-means method was used to allocate subjects into clusters in this procedure. The number of clusters must be determined by the researcher in the K-means method; no gold standard method has been established. Therefore, according to the ratio of between-cluster variance to within-cluster variance, cubic clustering criterion [19], the sample size, and the interpretation of each cluster, three clusters for each sex were considered to be appropriate in this study.

The median and interquartile ranges of the amount of food groups and nutrients were described for each cluster, men and women separately, because the distribution of food and iodine intake was considered to be skewed. Because iodine intake and excretion might be influenced by the intake of kelp and Japanese individuals consume kelp intermittently [10], the day-to-day variation in iodine intake and excretion is considered to be large. Therefore, habitual iodine intake should be assessed and it was calculated with the Best-Power method. The Best-Power method, in which the distribution is transformed to nearly normal and is adjusted for within-person variability, was proposed by Nusser et al. and details of the method have been mentioned elsewhere [20, 21]. The median differences in iodine intake and excretion among different clusters were examined using the Kruskal-Wallis test. The statistical difference in habitual iodine intake among clusters was not assessed because the statistical method, including the Best-Power method, was used for estimating the distribution of habitual intake [21]. To assess the prevalence of nutritional inadequacy in each dietary pattern, the percentage of participants who did not meet the recommendation for iodine (below the estimated average requirement (EAR) or over the UL) were calculated according to the EAR cut-point method [22]. The reference values of iodine according to the DRI were 95 μg/day for EAR and 3000 μg/day for UL. Comparison with the RDA (130 μg/day) was also calculated for reference. Considering the bioavailability of iodine, iodine excretion was compared with 90 % of the reference values in the DRI, because over 90 % of iodine was considered to be excreted in urine [11]. The statistical difference in percentages of inadequacy among groups was calculated with Fisher’s exact test. The percentage of participants with inadequate intake was compared with those within the normal range. All analyses were performed with SAS ver 9.4 (SAS Institute Inc., USA), including the Best-Power method. Two-sided p-values <0.05 were considered statistically significant.

## Results

Three different sets of clusters for dietary patterns were identified from the 195 men and three from the 195 women, as shown in Table 1. For men, the three clusters were labelled “Cluster I (Rice and vegetables),” “Cluster II (Meat, non-Japanese noodles, and sugar-sweetened beverages),” and “Cluster III (Fish, Japanese noodles, and alcohol).” The number of participants in each cluster was 101 for Cluster I, 34 for Cluster II, and 60 for Cluster III. The “Cluster I (Rice and vegetables)” pattern was mainly characterized by a high median intake of rice, miso soup, pulses, vegetables, and seaweed. The “Cluster II (Meat, non-Japanese noodles, and sugar-sweetened beverages)” pattern was characterized by a high median intake of pasta and Chinese noodles (the food group of “Non-Japanese noodles”), confectioneries, meat, and sugar-sweetened beverages. The “Cluster III (Fish, Japanese noodles, and alcohol)” pattern was characterized by a high median intake of Japanese noodles, pickled vegetables, fish, and alcoholic beverages. For women, three clusters, labelled “Cluster I (Rice and vegetables),” “Cluster II (Fish and Japanese noodles),” and “Cluster III (Bread and non-Japanese noodles),” could be identified. Among the 195 women, 22 were in Cluster I, 33 were in Cluster II, and 140 were in Cluster III. The “Cluster I (Rice and vegetables)” pattern was characterized by a high median intake of rice, miso soup, and vegetables. The “Cluster II (Fish and Japanese noodles)” pattern was characterized by a high median intake of Japanese noodles, pulses, fruit, seaweed, and fish. The “Cluster III (Bread and non-Japanese noodles)” pattern was characterized by a high median intake of pasta and Chinese noodles, bread, confectioneries, and sugar-sweetened beverages.

**Table 1:** Energy adjusted intake (g/1000 kcal) of 31 food groups across the three clusters (195 men and 195 women living in Japan)^a^

	Men						Women					
Food group	Cluster I (Rice and vegetables)		Cluster II (Meat, non-Japanese noodles, and sugar sweetened beverages)		Cluster III (Fish, Japanese noodles, and alcohol)		Cluster I (Rice and vegetables)		Cluster II (Fish and Japanese noodles)		Cluster III (Bread and non-Japanese noodles)	
	(*n* = 101)		(*n* = 34)		(*n* = 60)		(*n* = 22)		(*n* = 33)		(*n* = 140)	
	Median	IQR	Median	IQR	Median	IQR	Median	IQR	Median	IQR	Median	IQR
Rice	175	(146, 221)	148	(97, 183)	153	(111, 178)	155	(106, 202)	130	(101, 160)	138	(108, 172)
Miso soup	7	(2, 34)	2	(0, 5)	3	(0, 6)	17	(3, 46)	5	(2, 12)	5	(1, 16)
Japanese noodles	7	(0, 38)	0	(0, 23)	20	(0, 36)	0	(0, 17)	19	(0, 40)	0	(0, 20)
Non-Japanese noodles	12	(0, 34)	66	(17, 117)	24	(1, 54)	10	(0, 58)	5	(0, 23)	21	(0, 53)
Bread	9	(0, 26)	0	(0, 13)	17	(0, 26)	8	(0, 17)	9	(0, 23)	14	(0, 24)
Potatoes	40	(23, 58)	28	(18, 59)	24	(14, 40)	35	(17, 56)	40	(14, 62)	35	(18, 54)
Nuts	0	(0, 1)	0	(0, 1)	0	(0, 1)	1	(0, 1)	1	(0, 2)	0	(0, 2)
Pulses	23	(14, 37)	11	(4, 20)	13	(7, 25)	35	(21, 55)	43	(35, 72)	17	(8, 31)
Sugar	5	(3, 8)	4	(3, 7)	5	(3, 8)	7	(4, 10)	6	(5, 10)	6	(4, 9)
Confectionaries	10	(2, 19)	19	(13, 34)	18	(7, 29)	24	(11, 36)	25	(14, 34)	25	(14, 39)
Fat	0	(0, 1)	1	(0, 1)	1	(0, 2)	0	(0, 1)	0	(0, 1)	1	(0, 2)
Oil	8	(5, 10)	8	(7, 11)	6	(5, 10)	7	(4, 9)	8	(5, 9)	9	(6, 11)
Fruits	15	(6, 42)	2	(0, 9)	17	(1, 37)	32	(12, 46)	56	(26, 75)	19	(7, 45)
Green and Yellow vegetables	36	(26, 49)	21	(12, 25)	23	(13, 35)	63	(43, 91)	46	(37, 64)	32	(20, 49)
White vegetables	70	(55, 87)	43	(29, 58)	56	(41, 68)	117	(94, 143)	96	(84, 111)	62	(47, 83)
Pickled vegetables	2	(0, 5)	1	(0, 5)	3	(1, 8)	2	(0, 4)	9	(1,14)	2	(0, 5)
Mushrooms	6	(3, 11)	2	(1, 4)	3	(1, 6)	20	(11, 29)	9	(4, 15)	5	(2, 9)
Seaweeds	3	(1, 6)	1	(0, 2)	2	(0, 5)	4	(1, 7)	9	(5, 13)	2	(1, 5)
Fish	33	(19, 44)	15	(7, 21)	41	(30, 54)	37	(14, 51)	55	(42, 61)	23	(14, 36)
Fish products	5	(2, 10)	2	(0, 5)	10	(5,19)	5	(1, 11)	18	(12, 26)	6	(2, 11)
Meat	43	(31, 65)	44	(31, 70)	34	(21, 45)	40	(20, 57)	21	(15, 30)	35	(26, 46)
Meat products	5	(1, 10)	7	(4, 11)	4	(1, 6)	3	(0, 6)	3	(1, 6)	4	(2, 9)
Eggs	18	(13, 24)	10	(7, 16)	18	(12,22)	13	(8, 22)	20	(14, 29)	18	(10, 26)
Dairy products	32	(8, 68)	26	(10, 58)	20	(4, 46)	32	(14, 49)	74	(48, 100)	44	(20, 81)
Alcoholic beverages	4	(2, 70)	8	(1, 103)	105	(42, 209)	4	(2, 21)	3	(2, 9)	3	(1, 30)
Fruit and vegetable juice	0	(0, 9)	0	(0, 0)	0	(0, 0)	0	(0, 8)	0	(0, 0)	0	(0, 12)
Green tea	177	(60, 288)	168	(88, 241)	133	(36, 263)	236	(111, 421)	185	(130, 369)	190	(114, 338)
Tea	0	(0, 12)	0	(0, 0)	0	(0, 0)	14	(0, 72)	0	(0, 19)	0	(0, 25)
Coffee	92	(0, 159)	43	(0, 98)	152	(88, 239)	88	(0, 197)	177	(81, 265)	130	(35, 223)
Sugar sweetened beverages	0	(0, 7)	42	(6, 106)	0	(0, 14)	0	(0, 24)	0	(0,15)	6	(0, 34)
Seasonings	27	(18, 41)	23	(16, 35)	19	(15, 29)	49	(36, 67)	21	(18, 34)	23	(16, 34)

*IQR* interquartile range

^a^The intake of each food group was calculated from 4-day dietary records

The highest median values among the three clusters are underlined

The basic characteristics of participants in each cluster are shown in Tables 2. As for the dietary patterns of men, participants who belonged to the “Cluster II (Meat, non-Japanese noodles, and sugar-sweetened beverages)” pattern were more likely to be young (especially in their twenties), current smokers, and living alone. For women, participants with the “Cluster II (Fish and Japanese noodles)” pattern were more likely to be old and were shorter than participants in other groups. Table 3 and 4 show the median of iodine intake assessed with 4-day DR, habitual iodine intake calculated with Best-Power method, and iodine excretion from 24-hour urine collection across three clusters in 195 men and 195 women. For men, median iodine intake was the significantly lowest in “Cluster II (Meat, non-Japanese noodles, and sugar sweetened beverages)” and iodine excretion was also the lowest in that cluster but not statistically significant. For women, “Cluster III (Bread and non-Japanese noodles)” had the significantly lowest median intake and excretion of iodine.

**Table 2:** Basic characteristics across three dietary patterns identified among 195 Japanese men and 195 women^a^

		Men					Women				
	All	All men	Cluster I (Rice and vegetables)	Cluster II (Meat, non-Japanese noodles, and sugar sweetened beverages)	Cluster III (Fish, Japanese noodles, and alcohol)	*p*-value†	All women	Cluster I (Rice and vegetables)	Cluster II (Fish and Japanese noodles)	Cluster III (Bread and non-Japanese noodles)	*p*-value†
	(*n* = 390)	(*n* = 195)	(*n* = 101)	(*n* = 34)	(*n* = 60)		(*n* = 195)	(*n* = 22)	(*n* = 33)	(*n* = 140)	
Age (years)											
Mean ± SD	44.5 ± 13.4	44.7 ± 13.3	45.6 ± 13.0	35.2 ± 11.0	48.6 ± 12.4	<0.001	44.3 ± 13.4	43.3 ± 15.2	55.2 ± 9.81	41.9 ± 12.7	<0.001
Age class (number [%])											
20–29	74 (19.0)	36 (18.5)	13 (12.9)	16 (47.1)	7 (11.7)		38 (19.5)	7 (31.8)	1 (3.0)	30 (21.4)	
30–39	81 (20.8)	42 (21.5)	29 (28.7)	6 (17.7)	7 (11.7)		39 (20.0)	3 (13.6)	1 (3.0)	35 (25.0)	
40–49	79 (20.3)	38 (19.5)	17 (16.8)	7 (20.6)	14 (23.3)		41 (21.0)	4 (18.1)	6 (18.1)	31 (22.1)	
50–59	77 (19.7)	38 (19.5)	17 (16.8)	4 (11.8)	17 (28.3)		39 (20.0)	3 (13.6)	11 (33.3)	25 (17.9)	
60–69	79 (20.3)	41 (21.0)	25 (24.8)	1 (2.9)	15 (25.0)		38 (20.0)	5 (22.7)	14 (42.4)	19 (13.6)	
Body height (cm)	164.0 ± 8.4	170.3 ± 5.4	170.0 ± 5.6	171.1 ± 4.6	170.2 ± 5.5	0.62	157.6 ± 5.7	157.3 ± 6.6	155.5 ± 6.0	158.2 ± 5.4	0.05
Body weight (kg)	62.9 ± 12.6	69.7 ± 11.3	70.0 ± 11.4	69.9 ± 11.3	69.1 ± 11.4	0.88	56.1 ± 10.0	54.8 ± 8.3	57.5 ± 14.4	55.9 ± 8.9	0.59
Body Mass Index (kg/m^2^)	23.3 ± 3.6	24.0 ± 3.5	24.1 ± 3.3	23.9 ± 3.9	23.8 ± 3.6	0.83	22.6 ± 3.7	22.1 ± 3.2	23.6 ± 4.7	22.4 ± 3.4	0.17
Smoking status (number [%])											
Current smoker	101 (25.9)	73 (37.4)	34 (33.7)	19 (55.9)	20 (33.3)	0.04	28 (14.4)	1 (4.6)	2 (6.1)	25 (17.9)	0.13
Past smoker	71 (18.2)	57 (29.2)	27 (26.7)	6 (17.7)	24 (40.0)		14 (7.2)	0	3 (9.1)	11 (7.9)	
Non smoker	218 (55.9)	65 (33.3)	40 (39.6)	9 (26.5)	16 (26.7)		153(78.5)	21 (95.5)	28 (84.9)	104 (74.3)	
Living status (number [%])											
Alone	25 (6.4)	13 (6.7)	7 (6.9)	4 (11.8)	2 (3.3)	0.009	12 (6.2)	1 (4.6)	4 (12.1)	7 (5.0)	0.12
With family	347 (88.8)	173 (88.7)	90 (89.1)	25 (73.5)	58 (96.7)		174 (89.3)	18 (81.8)	28 (84.9)	128 (91.4)	
With others	18 (4.6)	9 (4.6)	4 (4.0)	5 (14.7)	0		9 (4.6)	3 (13.6)	1 (3.0)	5 (3.6)	
Past history or current treatment (number [%])											
Hypertension	47 (12.0)	27 (13.9)	11 (10.9)	4 (11.8)	12 (20.0)		20 (10.3)	3 (13.6)	7 (21.2)	10 (7.1)	
Hyperlipidemia	36 (9.2)	16 (8.2)	11 (10.9)	2 (5.9)	3 (5.0)		20 (10.3)	3 (13.6)	9 (27.3)	8 (5.7)	
Hyperuricemia	9 (2.3)	8 (4.1)	3 (3.0)	1 (2.9)	4 (6.7)		1 (0.5)	0	0	1 (0.7)	
Diabetes mellitus	8 (2.1)	6 (3.1)	3 (3.0)	1 (2.9)	2 (3.3)		2 (1.0)	0	2 (6.1)	0	
Renal dysfunction	1 (0.3)	1 (0.5)	0	0	1 (1.7)		0	0	0	0	
Medication (number [%])											
Diuretics	4 (1.0)	4 (2.1)	2 (2.0)	0	2 (3.3)		0	0	0	0	
Laxative	5 (1.3)	0	0	0	0		5 (2.6)	0	1 (3.0)	4 (2.9)	
Antibiotics	18 (4.6)	9 (4.6)	3 (3.0)	2 (5.9)	4 (6.7)		9 (4.6)	1 (4.6)	2 (6.1)	6 (4.3)	

*SD* standard deviation

^a^Values are mean ± SD or number of subjects. Percentage of subjects is in brackets

†To test statistical differences among clusters, analysis of variance (ANOVA) was used for continuous variables and the Chi-square test was used for categorical variables. For smoking and living status, Fisher’s exact test was used to test statistical differences. *P*-values for past history or medication were not calculated because of the limited number of subjects in each cluster

**Table 3:** Median of iodine intake and excretion across three dietary patterns identified by cluster analysis (*n* = 195, Japanese men)^a^

	All			Cluster I (Rice and vegetables)			Cluster II (Meat, non-Japanese noodles, and sugar sweetened beverages)			Cluster III (Fish, Japanese noodles, and alcohol)			*p*-value†
	n	Median	IQR	n	Median	IQR	n	Median	IQR	n	Median	IQR	
Total energy intake (kcal/d)	195	2357	2053, 2654	101	2298	2040, 2540	34	2372	2004, 2704	60	2400	2090, 2762	0.22
Crude iodine intake (μg/d)	195	632	210, 2025	101	1068	245, 2636	34	279	119, 1028	60	577	224, 1692	0.005
Habitual iodine intake (μg/d)	195	698	396, 1310	101	907	501, 1548	34	335	231, 535	60	625	397, 1068	-
Iodine excretion (μg/d)	179	417	203, 1297	95	409	203, 1448	30	344	183, 898	54	467	214, 1014	0.67
Iodine excretion (μg/gCre/d)	179	268	128, 817	95	267	134, 1034	30	201	109, 496	54	359	146, 796	0.41

*IQR* interquartile range

^a^Iodine intake was assessed with 4-day dietary records and iodine excretion by 24-h urine collection. Habitual iodine intake was calculated using the Best-Power method

† The Kruskal-Wallis test was used to test the median differences among clusters

**Table 4:** Median of iodine intake and excretion across three dietary patterns identified by cluster analysis (*n* = 195, Japanese women)^a^

	All			Cluster I (Rice and vegetables)			Cluster II (Fish and Japanese noodles)			Cluster III (Bread and non-Japanese noodles)			*p*-value†
	n	Median	IQR	n	Median	IQR	n	Median	IQR	n	Median	IQR	
Total energy intake (kcal/d)	195	1847	1654, 2097	22	1828	1719, 1943	33	1913	1665, 2096	140	1845	1634, 2124	0.62
Crude iodine intake (μg/d)	195	462	155, 2034	22	2376	204, 4070	33	1559	341, 5952	140	310	138, 1046	0.0002
Habitual iodine intake (μg/d)	195	511	282, 1080	22	1160	353, 4602	33	1400	852, 1829	140	374	247, 701	-
Iodine excretion (μg/d)	177	345	177, 978	21	581	405, 1481	28	472	241, 1374	128	306	171, 808	0.006
Iodine excretion (μg/gCre/d)	177	367	179, 931	21	741	393, 1409	28	486	302, 1220	128	311	166, 590	0.003

*IQR* interquartile range

^a^Iodine intake was assessed with 4-day dietary records and iodine excretion by 24-h urine collection. Habitual iodine intake was calculated using the Best-Power method

† The Kruskal-Wallis test was used to test the median differences among clusters

Table 5 shows the percentages of inadequate intake and excretion of iodine compared with the reference values in the DRI [11]. Although the number of participants in each cell was limited, the prevalence of insufficient iodine intake in “Cluster II (Meat, non-Japanese noodles, and sugar-sweetened beverages)” was the statistically significantly highest of the three clusters of men. In this Cluster II, the percentage of insufficient iodine excretion was higher and excess iodine intake and excretion was lower than in other clusters, but was not statistically significant. Excess iodine intake and excretion in “Cluster III (Bread and non-Japanese noodles)” was the statistically significantly lowest of the three clusters of women, and insufficient intake was high in this cluster but not statistically significant. In terms of the habitual iodine intake of men and women, hardly any participants were below the EAR level (<95 μg/d) and only 2 to 6 % of participants were below the RDA (<130 μg/d).

**Table 5:** Percentage of participants with inadequate iodine intake and excretion compared with the DRI for Japanese^a^

		Percentage of inadequacy in men (numbers)					Percentage of inadequacy in women (numbers)				
	Reference values†	All	Cluster I (Rice and vegetables)	Cluster II (Meat, non-Japanese noodles, and sugar sweetened beverages)	Cluster III (Fish, Japanese noodles, and alcohol)	*p*-value‡	All	Cluster I (Rice and vegetables)	Cluster II (Fish and Japanese noodles)	Cluster III (Bread and non-Japanese noodles)	*p*-value‡
			(*n* = 101)	(*n* = 34)	(*n* = 60)			(*n* = 22)	(*n* = 33)	(*n* = 140)	
Below EAR or RDA											
Iodine intake	<95 μg/d	7.7 (15)	5.9 (6)	23.5 (8)	1.7 (1)	0.003	15.9 (31)	13.6 (3)	6.1 (2)	18.6 (26)	0.39
	<130 μg/d	13.9 (27)	9.9 (10)	29.4 (10)	11.7 (7)	0.06	20.0 (39)	18.2 (4)	6.1 (2)	23.6 (33)	0.14
Habitual iodine intake	<95 μg/d	0.5 (1)	1.0 (1)	0 (0)	0 (0)	-	1.0 (2)	4.6 (1)	0 (0)	0.7 (1)	
	<130 μg/d	1.5 (3)	1.0 (1)	5.9 (2)	0 (0)	-	2.1 (4)	4.6 (1)	0 (0)	2.1 (3)	
Iodine excretion§	<95 μg/d	1.7 (3)	2.1 (2)	3.3 (1)	0 (0)	0.43	3.4 (6)	4.8 (1)	0 (0)	4.0 (5)	0.48
	<130 μg/d	4.5 (8)	3.2 (3)	6.7 (2)	5.6 (3)	0.69	9.0 (16)	4.8 (1)	3.6 (1)	10.9 (14)	0.61
Above UL											
Iodine intake	>3000 μg/d	18.5 (36)	22.8 (23)	5.9 (2)	18.3 (11)	0.18	21.5 (42)	50.0 (11)	36.4 (12)	13.6 (19)	0.0002
Habitual iodine intake	>3000 μg/d	5.1 (11)	8.9 (9)	0 (0)	3.3 (2)	-	7.2 (15)	36.4 (8)	15.2 (5)	1.4 (2)	-
Iodine excretion§	>3000 μg/d	8.4 (15)	8.4 (8)	6.7 (2)	9.3 (5)	1.0	4.0 (7)	14.3 (3)	7.1 (2)	1.6 (2)	0.01

Abbreviations: *DRI* dietary reference intakes 2015 for Japanese, *EAR* estimated average requirement, *RDA* recommended dietary allowance, *UL* tolerable upper intake level

^a^Intakes were calculated with 4-day dietary records. Habitual iodine intake was calculated using the Best-Power method in each cluster

† Reference values are according to the Dietary Reference Intakes 2015 for Japanese (Iodine: EAR is 95 μg/day, RDA is 130 μg/day, and UL is 3000 μg/day). Considering the bioavailability of iodine, iodine excretion was compared with 90 % of the reference values in the DRI [11]

‡ Fisher’s exact test was used to test the differences between the percentage of inadequacy and the percentage of participants within the normal intake range across the three clusters. The statistical difference in habitual iodine intake was not calculated because the statistical modelling method was applied for each cluster

§The number of participants in each cluster was 95 in Cluster I, 30 in Cluster II, and 54 in Cluster III for men and 21 in Cluster I, 28 in Cluster II, and 128 in Cluster III for women

## Discussion

This is the first study that examines inadequate iodine intake in the Japanese population in terms of dietary patterns derived from cluster analysis. We identified three dietary patterns, labelled “Cluster I (Rice and vegetables)” (*n* = 101), “Cluster II (Meat, non-Japanese noodles, and sugar-sweetened beverages)” (*n* = 34), and “Cluster III (Fish, Japanese noodles, and alcohol)” (*n* = 60), among 195 Japanese men. We identified three other dietary patterns among 195 Japanese women: Cluster I (Rice and vegetables)” (*n* = 22), “Cluster II (Fish and Japanese noodles)” (*n* = 33), and “Cluster III (Bread and non-Japanese noodles)” (*n* = 140). The men in “Cluster II (Meat, non-Japanese noodles, and sugar-sweetened beverages)” and the women in “Cluster III (Bread and non-Japanese noodles)” had a higher possibility of consuming an insufficient amount of iodine than participants in other clusters.

The nutritional adequacy of dietary patterns was examined in several studies from Western countries and from Japan [3–6]. Two previous Japanese studies which assessed the nutritional adequacy of four dietary patterns in young Japanese women and three patterns in pregnant women identified the “Rice” pattern (this included fish and vegetables in one study and derived a separate “Fish and vegetables” pattern in the other), the “Meat and eggs” pattern, and the “Wheat products” pattern (including bread and confectionaries) [5, 6]. Because of the different dietary assessment methods (DR in our study vs. a self-administered diet history questionnaire in the previous studies) and the difference in food grouping, a pattern that was characterized by Japanese noodles and fish was identified in both men and women in our study. We divided noodles into Japanese noodles and other non-Japanese noodles such as pasta because of the difference in the amount of iodine each contains. The soup served with Japanese noodles is mainly made of soup stock made from kelp and bonito—this is one of the main sources of iodine in the Japanese population [10]. Japanese noodles are usually eaten instead of rice and miso soup. Therefore, the derived clusters in the present study are interpretable and compatible with the style of current Japanese meals, and the dietary patterns characterized mainly by rice or wheat products were largely consistent with previous studies [5, 6].

As for iodine, only one study using principal component analysis from a Mediterranean country has evaluated the inadequacy of iodine status in dietary patterns [4]. Higher adherence to a “Mediterranean” dietary pattern, which is characterized by a higher intake of vegetables and fish, resulted in lower prevalence of inadequate (insufficient) iodine intake, while higher adherence to a “Western” dietary pattern, which is characterized by a higher intake of red meat and eggs, resulted in a higher prevalence of inadequate iodine intake. Although dairy products are one of the main sources of iodine in Western countries [23, 24], a high intake of low-fat dairy was observed in the “Mediterranean” pattern and whole-fat dairy was found in the “Western” pattern in this previous study. The median of dairy products was the highest in “Cluster I (Rice and vegetables)” in men and “Cluster II (Fish and Japanese noodles)” in women in this study; however, dairy products are not considered to be a main source of iodine in Japan [10]. Dietary sources of iodine in the Japanese population are considerably different from those of Western countries. Kelp and soup stock made from kelp accounted for nearly 90 % of the iodine intake in the Japanese population [10]. This was consistent with the result that the “Rice and vegetables” pattern and the “Fish and Japanese noodles” pattern in both men and women resulted in a high intake of iodine in our study. Although sources are different among countries, high iodine intake in these clusters might suggest that the spread of Japanese dishes which were included in these clusters may contribute to increase iodine intake of populations with iodine deficiency albeit only slightly. Moreover, temporary excess iodine intake may occur when people consume Japanese food including high amount of seaweed and soup stock unintentionally, although further study is required for more detailed information.

The median iodine intake in the “Cluster II (Meat, non-Japanese noodles, and sugar-sweetened beverages)” pattern in men and the “Cluster III (Bread and non-Japanese noodles)” pattern in women, was significantly lower than that for the other two dietary patterns. Younger Japanese participants had a lower iodine intake than older ones [10], and young participants were predominant in the “Cluster II (Meat, non-Japanese noodles, and sugar-sweetened beverages)” pattern in men and the “Cluster III (Bread and non-Japanese noodles)” pattern in women. Compared to the older generation, the dietary pattern in younger Japanese people is changing from typical Japanese patterns that mainly involve rice or Japanese noodles and contain iodine-rich foods. The prevalence of insufficient iodine intake might increase in the future and should be considered in Japan. In the previous studies, the habitual iodine intake of younger Japanese individuals was around 650 μg/d [10], and the iodine intake of Japanese students who did not consume seaweed on the survey day was 70 to 130 μg/d [13]. The calculated habitual iodine intake of subjects with the “Cluster II (Meat, non-Japanese noodles, and sugar-sweetened beverages)” pattern in men and the “Cluster III (Bread and non-Japanese noodles)” pattern in women was 335 μg/d and 374 μg/d, respectively. Despite the fact that only 2 to 6 % of participants were under the RDA (<130 μg/d) and only a few were below the EAR (<95 μg/d) in these groups, more attention should be given to the low intake group in the future.

The median of urinary iodine in the “Cluster II (Meat, non-Japanese noodles, and sugar-sweetened beverages)” pattern in men was lower than that in other patterns but the difference was not statistically significant. Although a large day-to-day variation in urinary iodine existed, we were not able to obtain multiple urine collection samples in this survey. The limited number of participants and survey days might have caused the non-significant results. Further research with multiple urine measurements is needed. As for the excessive intake, because 5 to 10 % of participants habitually consume iodine in an amount exceeding the UL level, excess iodine intake in Japanese individuals, especially those with the “Rice and vegetables” pattern and the “Japanese noodles and fish” pattern, should be noted. The cut-off value at which excess iodine intake may cause a clinical effect in Japanese individuals has not yet been clarified, and should also be researched in the future.

Several study limitations warrant mention. First, participants were co-workers of research dietitians and not a random sample. It is possible that these individuals may be more health-conscious than the average population. Nevertheless, the participants’ height and weight did not differ greatly from that of the general population in the national survey [25], and the survey areas were located throughout Japan, which increased the generalizability of the survey population. Second, the DR was limited to four days and only one urine sample was obtained for each participant. Number of days of the DR in this survey was originally determined to assess sodium intake in this survey. The large day-to-day variation in iodine intake due to the intermittent intake of kelp in the Japanese was recognized. For example, from the 16-day DR in the previous iodine study [10], although seaweed was consumed almost every day, kelp intake occurred once in 8 days on average and iodine intake above the UL (>3000 μg/d) occurred once in 4 to 5 days (data not shown). Therefore, the data from a limited number of survey days might not include a sufficient number of intermittent high iodine consumption days. Moreover, the discrepancy in iodine intake and excretion over the bioavailability of 90 % in this study may result from the difference in the number of survey days for DR and urine samples. Therefore, although habitual iodine intake was also estimated statistically, DR of more days and multiple urine measurements are needed to avoid random error, but were not feasible in this survey. Third, the sample size was originally determined for evaluating sodium intake and not for iodine. The number of subjects who underwent DR was limited to half of the total participants due to the feasibility of the survey, and one of the clusters only included 22 participants. This small number of subjects might decrease the statistical power. However, this is the first study to show the iodine status of Japanese individuals with several dietary patterns with objective urine data and it may be useful for future iodine studies and nutritional education. Further research that includes more participants is needed. Fourth, misreporting in the DR could not be completely avoided. Misreporting of food weight, especially when participants ate outside of the home and did not know the concentration of soup stock and its source, would have caused incorrect estimations in nutrient intake. To minimize the inaccuracy, we asked participants to obtain the names and menus of restaurants and estimated weights as much as possible. Because of variation in the iodine content of kelp, and in the concentration of soup stock (amount of iodine eluted from kelp into soup stock) could not be precisely recorded so iodine intake calculated from DR might lack accuracy. In addition, other sources of iodine such as povidone iodine were not assessed in this survey. Since one study reported that gargle containing povidone iodine affected urinary iodine excretion in Japanese [26], non-dietary sources of iodine should be assessed in further studies. These might be the reasons for the different values between iodine intake from DR and excretion from urine samples. To examine this, the correlation between iodine intake from DR and urinary iodine excretion was calculated. When DR and urinary samples were obtained on the same day in this dataset, the calculated iodine intake and urinary iodine excretion were significantly correlated (r = 0.37, *p* = 0.005). This result supported the accuracy of this calculation method of iodine to some extent. Nevertheless, further research that investigates the accuracy of iodine estimation is needed.

## Conclusion

The adequacy of iodine intake in three dietary patterns was examined using DR and 24-hour urine collection in Japanese men and women for the first time. People with dietary patterns that differed from the traditional Japanese diet and were unlikely to include iodine-rich foods consumed significantly low amounts of iodine. This pattern was mainly observed in younger participants. Therefore, although the habitual iodine intake of almost all participants was above the EAR and Japan is known to be a high iodine-consuming country, iodine insufficiency may gradually increase in the future with the change in dietary patterns. Further studies to identify subgroups with low iodine intake and to examine the association between iodine inadequacy (both excess and insufficient intake) and clinical symptoms are required.

## Additional file

Additional file 1:
**31 food groups used in cluster analysis.** (CSV 6 kb)

## Abbreviations

BMI: Body mass index
DR: Diet records
DRI: Dietary Reference Intakes 2015 for Japanese
EAR: Estimated average requirement
RDA: Recommended dietary allowance
UL: Tolerable upper intake level
